# 624 Implementation of a Collaborative Drug Therapy Management Protocol in an Adult Burn Clinic

**DOI:** 10.1093/jbcr/irac012.252

**Published:** 2022-03-23

**Authors:** Allison N Boyd, Todd A Walroth, Brett C Hartman

**Affiliations:** Eskenazi Health, Indianapolis, Indiana; Eskenazi Health, Indianapolis, Indiana; Richard M. Fairbanks Burn Center, Indianapolis, Indiana

## Abstract

**Introduction:**

Collaborative Drug Therapy Management (CDTM) protocols allow qualified clinical pharmacists working within a defined context to independently assume responsibility for a variety of direct patient care activities. The goal of this model is to improve access to care, often in a more affordable and timely manner. Pharmacists in our burn center have historically assisted with discharge planning and transitions of care for patients being sent home or to a facility. Despite these efforts, pharmacists were not formally involved in managing Burn Clinic patients. The objective of implementing a CDTM Protocol in Burn Clinic was to streamline management of burn-related pharmacologic issues.

**Methods:**

The CDTM protocol allows the pharmacist to assess patients’ therapeutic needs related to thermal injury, inhalation injury, or dermatologic disorder. Disease states managed include: pain, agitation, delirium, insomnia, venous thromboembolism, skin and soft tissue infections, and complications of hypermetabolic burn response.

**Results:**

In Burn Clinic, via in-person or telephone visits, pharmacists can independently evaluate and modify pharmacologic treatment regimens in accordance with state legislation, including initiation, renewal, and adjustment of drugs and vitamins. The pharmacist can also review, order, interpret, and conduct pertinent laboratory studies and wound culture results. In addition, the pharmacist completes medication reconciliation, performs patient education, and consults other healthcare providers, as needed. All visits, patient care, education, and treatment decisions are documented in the electronic record. “Incident-to” billing is completed at Level 99211, as this is a hospital-based clinic.

**Conclusions:**

Historically, pharmacists were peripherally involved with Burn Clinic patients when issues arose, serving in an “as-needed” capacity. With more proactive involvement, pharmacists are uniquely positioned to help with de-prescribing, drug interactions, insurance issues, access to medications, adherence, and dose optimization. Implementing a CDTM protocol allows pharmacists to become more formally involved in post-discharge follow-up and to help manage ambulatory burn patients.

